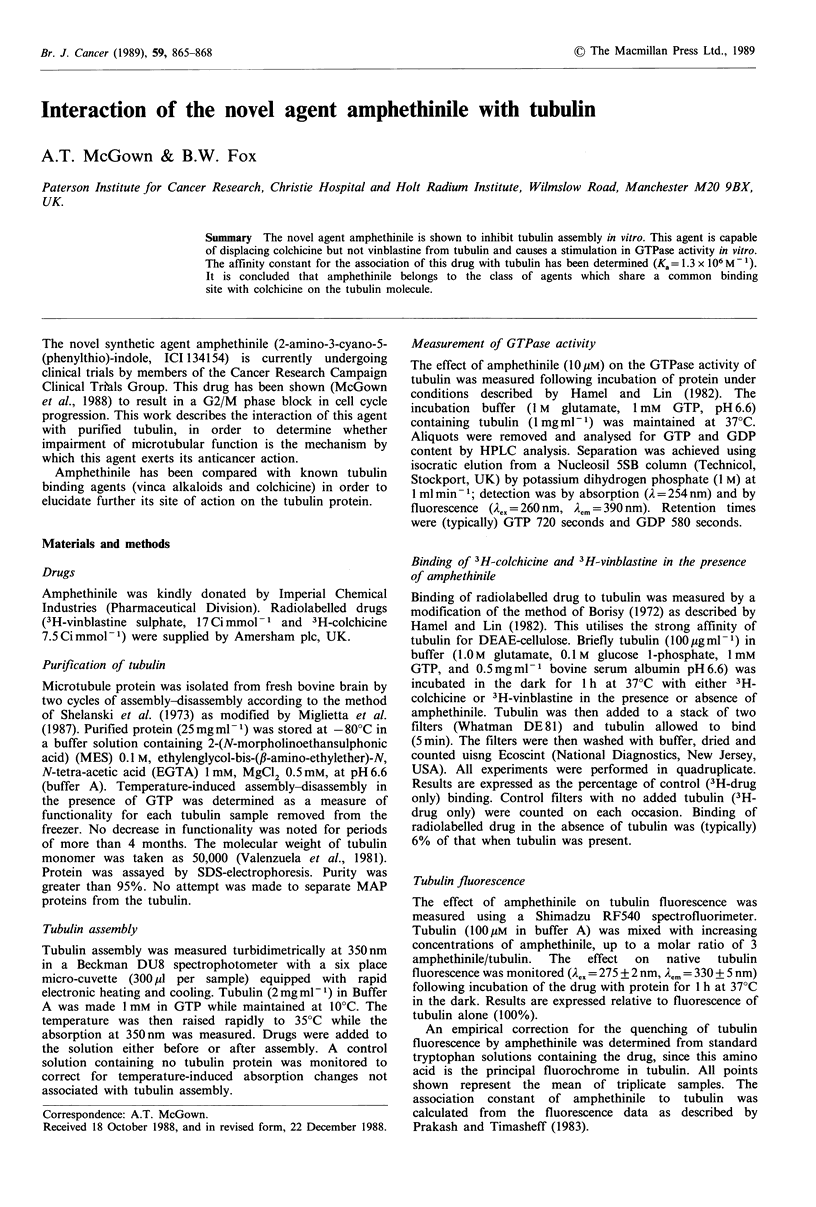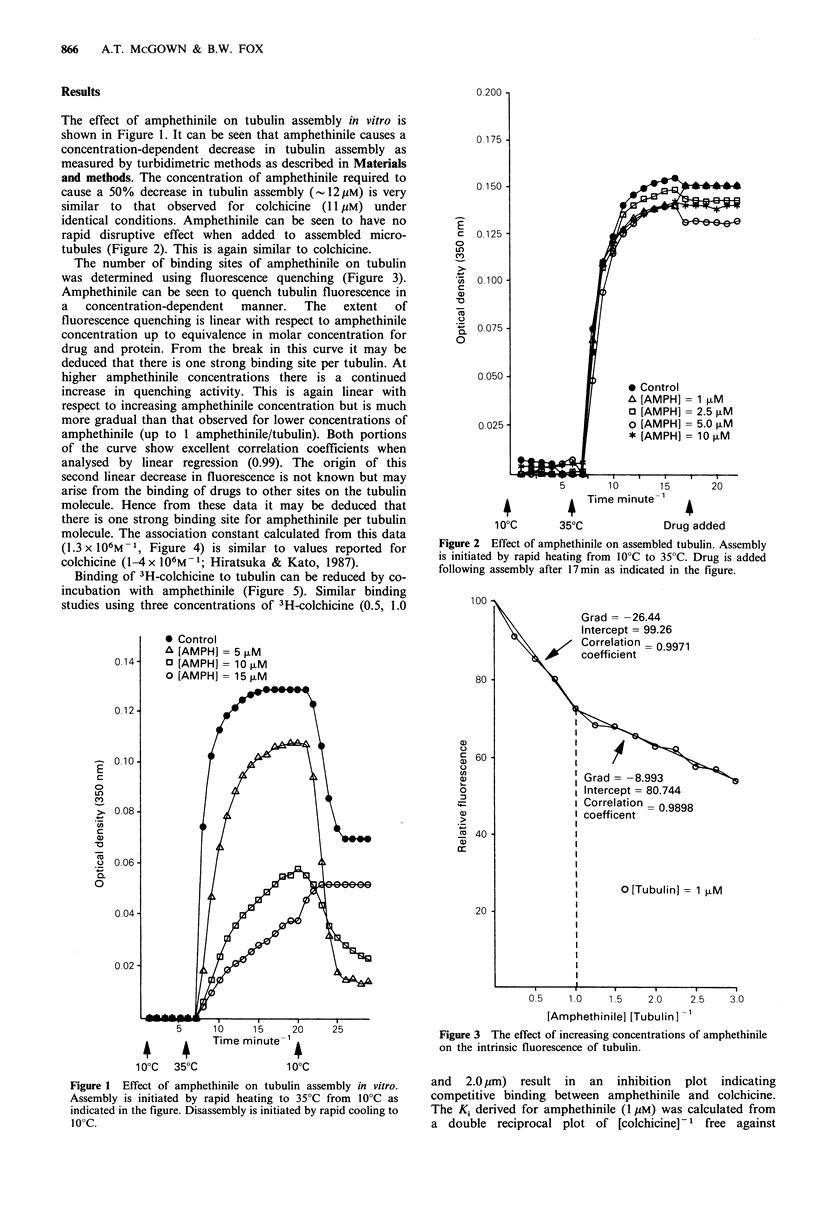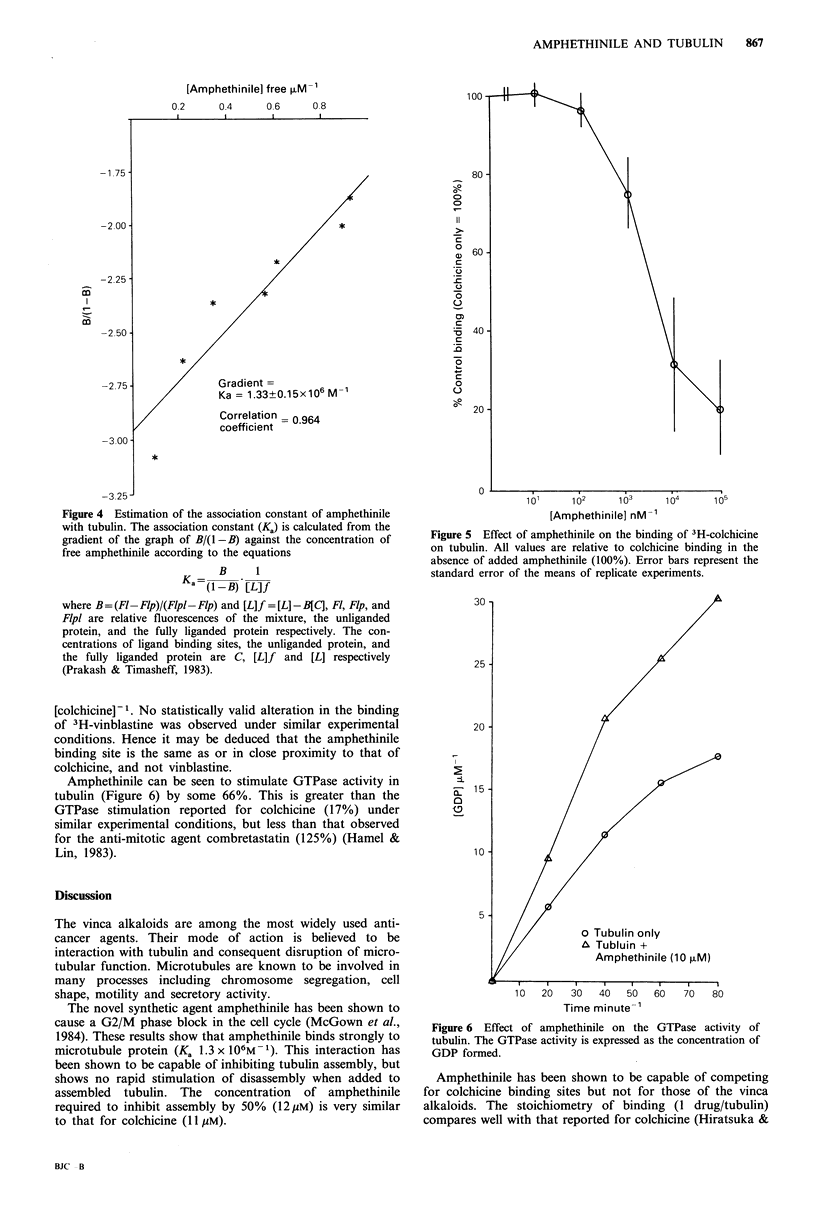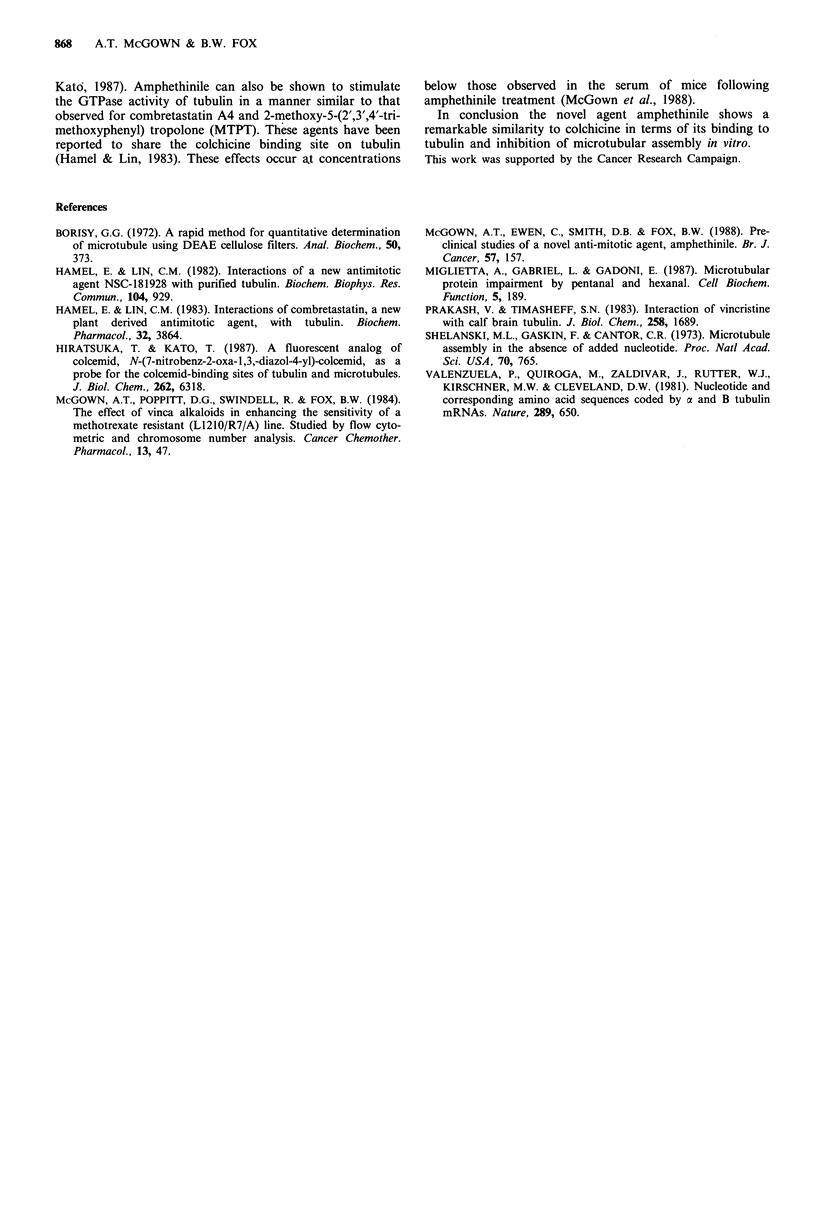# Interaction of the novel agent amphethinile with tubulin.

**DOI:** 10.1038/bjc.1989.183

**Published:** 1989-06

**Authors:** A. T. McGown, B. W. Fox

**Affiliations:** Paterson Institute for Cancer Research, Christie Hospital and Holt Radium Institute, Manchester, UK.

## Abstract

The novel agent amphethinile is shown to inhibit tubulin assembly in vitro. This agent is capable of displacing colchicine but not vinblastine from tubulin and causes a stimulation in GTPase activity in vitro. The affinity constant for the association of this drug with tubulin has been determined (Ka = 1.3 x 10(6) M-1). It is concluded that amphethinile belongs to the class of agents which share a common binding site with colchicine on the tubulin molecule.


*Br. J. Cancer* (1989), **59**, 865–868 © The Macmillan Press Ltd., 1989

### Interaction of the novel agent amphethinile with tubulin

A.T. McGown & B.W. Fox

*Paterson Institute for Cancer Research, Christie Hospital and Holt Radium Institute, Wilmslow Road, Manchester M20 9BX, UK.*

**Summary** The novel agent amphethinile is shown to inhibit tubulin assembly *in vitro*. This agent is capable of displacing colchicine but not vinblastine from tubulin and causes a stimulation in GTPase activity *in vitro*. The affinity constant for the association of this drug with tubulin has been determined ($K_a = 1.3 \times 10^6\,M^{-1}$). It is concluded that amphethinile belongs to the class of agents which share a common binding site with colchicine on the tubulin molecule.

The novel synthetic agent amphethinile (2-amino-3-cyano-5-(phenylthio)-indole, ICI 134154) is currently undergoing clinical trials by members of the Cancer Research Campaign Clinical Trials Group. This drug has been shown (McGown *et al.*, 1988) to result in a G2/M phase block in cell cycle progression. This work describes the interaction of this agent with purified tubulin, in order to determine whether impairment of microtubular function is the mechanism by which this agent exerts its anticancer action.

Amphethinile has been compared with known tubulin binding agents (vinca alkaloids and colchicine) in order to elucidate further its site of action on the tubulin protein.

### Materials and methods

### *Drugs*

Amphethinile was kindly donated by Imperial Chemical Industries (Pharmaceutical Division). Radiolabelled drugs ($^3$H-vinblastine sulphate, 17 Ci mmol$^{-1}$ and $^3$H-colchicine 7.5 Ci mmol$^{-1}$) were supplied by Amersham plc, UK.

### *Purification of tubulin*

Microtubule protein was isolated from fresh bovine brain by two cycles of assembly–disassembly according to the method of Shelanski *et al.* (1973) as modified by Miglietta *et al.* (1987). Purified protein (25 mg ml$^{-1}$) was stored at $-80$°C in a buffer solution containing 2-(*N*-morpholinoethansulphonic acid) (MES) 0.1 M, ethylenglycol-bis-($\beta$-amino-ethylether)-*N*, *N*-tetra-acetic acid (EGTA) 1 mM, $MgCl_2$ 0.5 mM, at pH 6.6 (buffer A). Temperature-induced assembly–disassembly in the presence of GTP was determined as a measure of functionality for each tubulin sample removed from the freezer. No decrease in functionality was noted for periods of more than 4 months. The molecular weight of tubulin monomer was taken as 50,000 (Valenzuela *et al.*, 1981). Protein was assayed by SDS-electrophoresis. Purity was greater than 95%. No attempt was made to separate MAP proteins from the tubulin.

### *Tubulin assembly*

Tubulin assembly was measured turbidimetrically at 350 nm in a Beckman DU8 spectrophotometer with a six place micro-cuvette (300 $\mu$l per sample) equipped with rapid electronic heating and cooling. Tubulin (2 mg ml$^{-1}$) in Buffer A was made 1 mM in GTP while maintained at 10°C. The temperature was then raised rapidly to 35°C while the absorption at 350 nm was measured. Drugs were added to the solution either before or after assembly. A control solution containing no tubulin protein was monitored to correct for temperature-induced absorption changes not associated with tubulin assembly.

Correspondence: A.T. McGown.
Received 18 October 1988, and in revised form, 22 December 1988.

### *Measurement of GTPase activity*

The effect of amphethinile (10 $\mu$M) on the GTPase activity of tubulin was measured following incubation of protein under conditions described by Hamel and Lin (1982). The incubation buffer (1 M glutamate, 1 mM GTP, pH 6.6) containing tubulin (1 mg ml$^{-1}$) was maintained at 37°C. Aliquots were removed and analysed for GTP and GDP content by HPLC analysis. Separation was achieved using isocratic elution from a Nucleosil 5SB column (Technicol, Stockport, UK) by potassium dihydrogen phosphate (1 M) at 1 ml min$^{-1}$; detection was by absorption ($\lambda = 254$ nm) and by fluorescence ($\lambda_{ex} = 260$ nm, $\lambda_{em} = 390$ nm). Retention times were (typically) GTP 720 seconds and GDP 580 seconds.

### *Binding of $^3$H-colchicine and $^3$H-vinblastine in the presence of amphethinile*

Binding of radiolabelled drug to tubulin was measured by a modification of the method of Borisy (1972) as described by Hamel and Lin (1982). This utilises the strong affinity of tubulin for DEAE-cellulose. Briefly tubulin (100 $\mu$g ml$^{-1}$) in buffer (1.0 M glutamate, 0.1 M glucose 1-phosphate, 1 mM GTP, and 0.5 mg ml$^{-1}$ bovine serum albumin pH 6.6) was incubated in the dark for 1 h at 37°C with either $^3$H-colchicine or $^3$H-vinblastine in the presence or absence of amphethinile. Tubulin was then added to a stack of two filters (Whatman DE 81) and tubulin allowed to bind (5 min). The filters were then washed with buffer, dried and counted uisng Ecoscint (National Diagnostics, New Jersey, USA). All experiments were performed in quadruplicate. Results are expressed as the percentage of control ($^3$H-drug only) binding. Control filters with no added tubulin ($^3$H-drug only) were counted on each occasion. Binding of radiolabelled drug in the absence of tubulin was (typically) 6% of that when tubulin was present.

### *Tubulin fluorescence*

The effect of amphethinile on tubulin fluorescence was measured using a Shimadzu RF540 spectrofluorimeter. Tubulin (100 $\mu$M in buffer A) was mixed with increasing concentrations of amphethinile, up to a molar ratio of 3 amphethinile/tubulin. The effect on native tubulin fluorescence was monitored ($\lambda_{ex} = 275 \pm 2$ nm, $\lambda_{em} = 330 \pm 5$ nm) following incubation of the drug with protein for 1 h at 37°C in the dark. Results are expressed relative to fluorescence of tubulin alone (100%).

An empirical correction for the quenching of tubulin fluorescence by amphethinile was determined from standard tryptophan solutions containing the drug, since this amino acid is the principal fluorochrome in tubulin. All points shown represent the mean of triplicate samples. The association constant of amphethinile to tubulin was calculated from the fluorescence data as described by Prakash and Timasheff (1983).

### Results

The effect of amphethinile on tubulin assembly *in vitro* is shown in Figure 1. It can be seen that amphethinile causes a concentration-dependent decrease in tubulin assembly as measured by turbidimetric methods as described in **Materials and methods**. The concentration of amphethinile required to cause a 50% decrease in tubulin assembly ($\sim 12\,\mu$M) is very similar to that observed for colchicine ($11\,\mu$M) under identical conditions. Amphethinile can be seen to have no rapid disruptive effect when added to assembled microtubules (Figure 2). This is again similar to colchicine.

The number of binding sites of amphethinile on tubulin was determined using fluorescence quenching (Figure 3). Amphethinile can be seen to quench tubulin fluorescence in a concentration-dependent manner. The extent of fluorescence quenching is linear with respect to amphethinile concentration up to equivalence in molar concentration for drug and protein. From the break in this curve it may be deduced that there is one strong binding site per tubulin. At higher amphethinile concentrations there is a continued increase in quenching activity. This is again linear with respect to increasing amphethinile concentration but is much more gradual than that observed for lower concentrations of amphethinile (up to 1 amphethinile/tubulin). Both portions of the curve show excellent correlation coefficients when analysed by linear regression (0.99). The origin of this second linear decrease in fluorescence is not known but may arise from the binding of drugs to other sites on the tubulin molecule. Hence from these data it may be deduced that there is one strong binding site for amphethinile per tubulin molecule. The association constant calculated from this data ($1.3 \times 10^6$M$^{-1}$, Figure 4) is similar to values reported for colchicine ($1–4 \times 10^6$M$^{-1}$; Hiratsuka & Kato, 1987).

Binding of $^3$H-colchicine to tubulin can be reduced by co-incubation with amphethinile (Figure 5). Similar binding studies using three concentrations of $^3$H-colchicine (0.5, 1.0 and 2.0 $\mu$m) result in an inhibition plot indicating competitive binding between amphethinile and colchicine. The $K_i$ derived for amphethinile (1 $\mu$M) was calculated from a double reciprocal plot of [colchicine]$^{-1}$ free against

**Figure 1** Effect of amphethinile on tubulin assembly *in vitro*. Assembly is initiated by rapid heating to 35°C from 10°C as indicated in the figure. Disassembly is initiated by rapid cooling to 10°C.

**Figure 2** Effect of amphethinile on assembled tubulin. Assembly is initiated by rapid heating from 10°C to 35°C. Drug is added following assembly after 17 min as indicated in the figure.

**Figure 3** The effect of increasing concentrations of amphethinile on the intrinsic fluorescence of tubulin.

**Figure 4** Estimation of the association constant of amphethinile with tubulin. The association constant ($K_a$) is calculated from the gradient of the graph of $B/(1-B)$ against the concentration of free amphethinile according to the equations

$$K_a=\frac{B}{(1-B)}\cdot\frac{1}{[L]f}$$

where $B=(Fl-Flp)/(Flpl-Flp)$ and $[L]f=[L]-B[C]$, $Fl$, $Flp$, and $Flpl$ are relative fluorescences of the mixture, the unliganded protein, and the fully liganded protein respectively. The concentrations of ligand binding sites, the unliganded protein, and the fully liganded protein are $C$, $[L]f$ and $[L]$ respectively (Prakash & Timasheff, 1983).

**Figure 5** Effect of amphethinile on the binding of $^3$H-colchicine on tubulin. All values are relative to colchicine binding in the absence of added amphethinile (100%). Error bars represent the standard error of the means of replicate experiments.

**Figure 6** Effect of amphethinile on the GTPase activity of tubulin. The GTPase activity is expressed as the concentration of GDP formed.

[colchicine]$^{-1}$. No statistically valid alteration in the binding of $^3$H-vinblastine was observed under similar experimental conditions. Hence it may be deduced that the amphethinile binding site is the same as or in close proximity to that of colchicine, and not vinblastine.

Amphethinile can be seen to stimulate GTPase activity in tubulin (Figure 6) by some 66%. This is greater than the GTPase stimulation reported for colchicine (17%) under similar experimental conditions, but less than that observed for the anti-mitotic agent combretastatin (125%) (Hamel & Lin, 1983).

### Discussion

The vinca alkaloids are among the most widely used anticancer agents. Their mode of action is believed to be interaction with tubulin and consequent disruption of microtubular function. Microtubules are known to be involved in many processes including chromosome segregation, cell shape, motility and secretory activity.

The novel synthetic agent amphethinile has been shown to cause a G2/M phase block in the cell cycle (McGown *et al.*, 1984). These results show that amphethinile binds strongly to microtubule protein ($K_a$ $1.3\times10^6\text{M}^{-1}$). This interaction has been shown to be capable of inhibiting tubulin assembly, but shows no rapid stimulation of disassembly when added to assembled tubulin. The concentration of amphethinile required to inhibit assembly by 50% (12 μM) is very similar to that for colchicine (11 μM).

Amphethinile has been shown to be capable of competing for colchicine binding sites but not for those of the vinca alkaloids. The stoichiometry of binding (1 drug/tubulin) compares well with that reported for colchicine (Hiratsuka &

Kato, 1987). Amphethinile can also be shown to stimulate the GTPase activity of tubulin in a manner similar to that observed for combretastatin A4 and 2-methoxy-5-(2′,3′,4′-trimethoxyphenyl) tropolone (MTPT). These agents have been reported to share the colchicine binding site on tubulin (Hamel & Lin, 1983). These effects occur at concentrations below those observed in the serum of mice following amphethinile treatment (McGown *et al.*, 1988).

In conclusion the novel agent amphethinile shows a remarkable similarity to colchicine in terms of its binding to tubulin and inhibition of microtubular assembly *in vitro*.

This work was supported by the Cancer Research Campaign.

### References

BORISY, G.G. (1972). A rapid method for quantitative determination of microtubule using DEAE cellulose filters. *Anal. Biochem.*, **50**, 373.

HAMEL, E. & LIN, C.M. (1982). Interactions of a new antimitotic agent NSC-181928 with purified tubulin. *Biochem. Biophys. Res. Commun.*, **104**, 929.

HAMEL, E. & LIN, C.M. (1983). Interactions of combretastatin, a new plant derived antimitotic agent, with tubulin. *Biochem. Pharmacol.*, **32**, 3864.

HIRATSUKA, T. & KATO, T. (1987). A fluorescent analog of colcemid, *N*-(7-nitrobenz-2-oxa-1,3,-diazol-4-yl)-colcemid, as a probe for the colcemid-binding sites of tubulin and microtubules. *J. Biol. Chem.*, **262**, 6318.

McGOWN, A.T., POPPITT, D.G., SWINDELL, R. & FOX, B.W. (1984). The effect of vinca alkaloids in enhancing the sensitivity of a methotrexate resistant (L1210/R7/A) line. Studied by flow cytometric and chromosome number analysis. *Cancer Chemother. Pharmacol.*, **13**, 47.

McGOWN, A.T., EWEN, C., SMITH, D.B. & FOX, B.W. (1988). Preclinical studies of a novel anti-mitotic agent, amphethinile. *Br. J. Cancer*, **57**, 157.

MIGLIETTA, A., GABRIEL, L. & GADONI, E. (1987). Microtubular protein impairment by pentanal and hexanal. *Cell Biochem. Function*, **5**, 189.

PRAKASH, V. & TIMASHEFF, S.N. (1983). Interaction of vincristine with calf brain tubulin. *J. Biol. Chem.*, **258**, 1689.

SHELANSKI, M.L., GASKIN, F. & CANTOR, C.R. (1973). Microtubule assembly in the absence of added nucleotide. *Proc. Natl Acad. Sci. USA*, **70**, 765.

VALENZUELA, P., QUIROGA, M., ZALDIVAR, J., RUTTER, W.J., KIRSCHNER, M.W. & CLEVELAND, D.W. (1981). Nucleotide and corresponding amino acid sequences coded by α and B tubulin mRNAs. *Nature*, **289**, 650.